# 1106. Evaluation of Penetration of Cefiderocol into Cerebrospinal Fluid Using a Rat Meningitis Model

**DOI:** 10.1093/ofid/ofab466.1300

**Published:** 2021-12-04

**Authors:** Miki Takemura, Sachi Kanazawa, Naoki Kohira, Yuki Aoe, Atsushi Morimoto, Kana Horiuchi, Yuji Inoue, Yoshinori Yamano

**Affiliations:** 1 Shionogi & Co., Ltd., Osaka, Osaka, Japan; 2 Shionogi TechnoAdvance Research Co., Ltd., Toyonaka, Osaka, Japan

## Abstract

**Background:**

Central nervous system (CNS) infections caused by Gram-negative bacteria (GNB) are sometimes hard to treat due to antibiotic resistance and difficulty with penetration into cerebrospinal fluid (CSF). Cefiderocol (CFDC) which was approved by the FDA and the EMA in 2019 to 2020 is a siderophore cephalosporin with potent activity against various GNB including carbapenem-resistant strains. In this study, we evaluated the penetration of CFDC into CSF using a rat meningitis model.

**Methods:**

To induce meningitis, the anesthetized immunocompetent rats were infected by intracisternal inoculation of a bacterial suspension of 8.7×10^1^ CFU of *E. coli* SR200138. 200 mg/kg or 50 mg/kg of CFDC was administered via tail vein bolus injection to uninfected rats (n=4/sampling point) and rats with meningitis (n=4/sampling point) 24 hours after infection. CSF was collected by cisternal puncture and blood was collected from heart. The samplings were performed 0.25, 0.5, 1, 3, and 5 hours after dosing. The concentrations of CFDC in plasma and CSF for individuals were determined by LC/MS/MS. PK parameters for the average values in plasma and CSF were calculated.

**Results:**

CFDC concentration and the PK parameters are shown in Figure and Table, respectively. The penetration of CFDC from plasma to CSF was observed in both uninfected and meningitis groups, and the penetration rates increased in the rats withs meningitis (AUC_CSF_/AUC_plasma_: 0.149-0.183) compared with the uninfected rats (AUC_CSF_/AUC_plasma_: 0.0508-0.0588). The penetration rates of CFDC in the meningitis were comparable to those of piperacillin, cefepime, and meropenem in human (0.32, 0.103, and 0.39 in strongly inflamed meninges, respectively) [1]. In both groups, elimination of CFDC from CSF was slower compared with that from plasma as seen with other β-lactam antibiotics such as meropenem, suggesting that T_> MIC_, an indicator that correlates with the efficacy of β-lactams, may be higher in CSF [2].

Table. PK Parameters of Cefiderocol after Intravenous Bolus Administration in Uninfected Rats and Rats with Meningitis

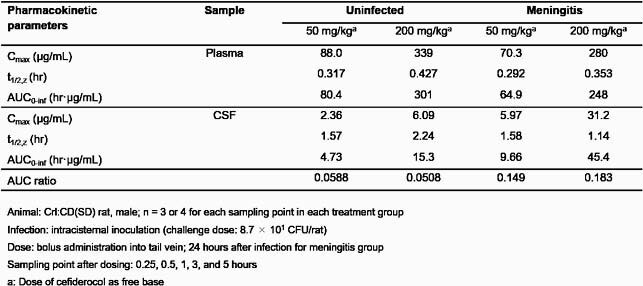

Figure. Concentrations of Cefiderocol after Intravenous Bolus Administration in Uninfected Rats and Rats with Meningitis

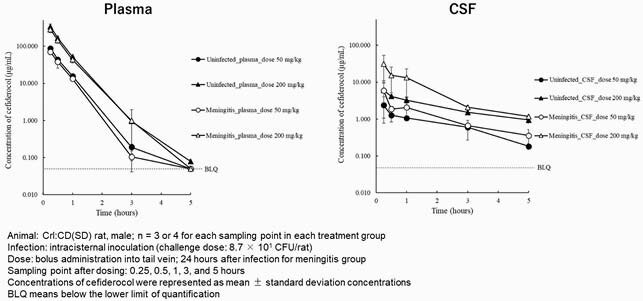

**Conclusion:**

It was confirmed that CFDC penetrates into CSF from plasma in a rat model and the penetration rate was increased 3-fold in meningitis.

**References:**

1. Nau, R. et al. Clin Microbiol Rev. 2010 Oct;23(4):858–883.

2. Nau, R. et al. Antimicrob Agents Chemother. 1998 Aug;42(8):2012–2016.

**Disclosures:**

**Miki Takemura, MS**, **SHIONOGI & CO., LTD.** (Employee) **Sachi Kanazawa, PhD**, **Shionogi & Co., Ltd.** (Employee) **Naoki Kohira, PhD**, **Shionogi & Co., Ltd.** (Employee) **Yuki Aoe, BS**, **Shionogi TechnoAdvance Research Co., Ltd.** (Employee) **Atsushi Morimoto, n/a**, **Shionogi TechnoAdvance Research Co., Ltd.** (Employee) **Kana Horiuchi, MPharm**, **Shionogi & Co., Ltd.** (Employee) **Yuji Inoue, MPharm**, **Shionogi & Co., Ltd.** (Employee) **Yoshinori Yamano, PhD**, **Shionogi** (Employee)

